# The relationship between cognitive and emotional representations of peripheral neuropathy and incident diabetes-related foot ulceration

**DOI:** 10.1186/1757-1146-4-S1-O37

**Published:** 2011-05-20

**Authors:** Byron Perrin, Hal Swerissen, Craig Payne

**Affiliations:** 1 La Trobe Rural Health School, La Trobe University, Bendigo, Victoria, 3550, Australia; 2 Musculoskeletal Research Centre, La Trobe University, Bundoora, Victoria, 3086, Australia; 3 Faculty of Health Sciences, La Trobe University, Bundoora, Victoria, 3086, Australia; 4 Department of Podiatry, La Trobe University. Bundoora, Victoria, 3086, Australia; 5 Bendigo Health, Bendigo, Victoria, 3550, Australia

## Background

The common sense model of illness (CSM) has been shown to be a useful model to help understand the psychological influences on diabetes-related behaviour and health outcomes. Using the CSM, the aim of this study was to investigate the relationship between cognitive and emotional representations of peripheral neuropathy and diabetes-related foot ulceration in adults with diabetes.

## Methods

One-hundred and twenty-one people with diabetes and peripheral neuropathy were recruited into this one-year prospective cohort study. At baseline, the participants completed two questionnaires- the Patients’ Interpretation of Neuropathy questionnaire and a short questionnaire asking about preventative foot-care behaviour. Basic diabetes and demographic information was also collected. Sequential logistic regression was used to investigate the influence of cognitive and emotional representations of peripheral neuropathy as measured by the PIN and the development of incident foot ulceration.

## Results

One-hundred and seventeen participants completed the study. The incidence of new foot ulceration was 34.2%. Only two statistically significant independent risk factors for foot ulceration were detected: prior history of foot ulceration (OR= 3.1; 95%CI: 1.16-8.18; p=.024) and severity of neuropathy (OR=1.1; 95%CI: 1.00-1.15; p=.047).

## Conclusions

A consistent association between cognitive and emotional representations of peripheral neuropathy and incident foot pathology was not found. If the CSM is to be clinically useful for people with diabetes and peripheral neuropathy the mediational role of preventative foot-care behaviour should be further investigated.

